# Uncovering Allele-Specific Expression Patterns Associated with Sea Lice (*Caligus rogercresseyi*) Burden in Atlantic Salmon

**DOI:** 10.3390/genes16070841

**Published:** 2025-07-19

**Authors:** Pablo Cáceres, Paulina López, Carolina Araya, Daniela Cichero, Liane N. Bassini, José M. Yáñez

**Affiliations:** 1 Facultad de Ciencias Veterinarias y Pecuarias, Universidad de Chile, Santiago 8820000, Chile; damp85@uchile.cl (P.C.); caaraya@uchile.cl (C.A.); 2 Center for Research and Innovation in Aquaculture (CRIA), Universidad de Chile, Santiago 8820808, Chile; 3 AquaGen Chile, Puerto Varas 5550000, Chile; paulina.lopez@aquagenchile.cl (P.L.); daniela.cichero@aquagenchile.cl (D.C.); 4 Escuela Medicina Veterinaria, Facultad de Ciencias de la Vida, Universidad Andres Bello, Santiago 8370251, Chile; liane.bassini@unab.cl; 5 Millennium Institute Center for Genome Regulation (CGR), Santiago 8320165, Chile

**Keywords:** sea lice, RNA-seq, DGE, ASE

## Abstract

Background/Objetives: Sea lice (*Caligus rogercresseyi*) pose a major threat to Atlantic salmon (*Salmo salar*) aquaculture by compromising fish health and reducing production efficiency. While genetic variation in parasite load has been reported, the molecular mechanisms underlying this variation remain unclear. Methods: two sea lice challenge trials were conducted, achieving high infestation rates (47.5% and 43.5%). A total of 85 fish, selected based on extreme phenotypes for lice burden (42 low, 43 high), were subjected to transcriptomic analysis. Differential gene expression was integrated with allele-specific expression (ASE) analysis to uncover cis-regulatory variation influencing host response. Results: Sixty genes showed significant ASE (*p* < 0.05), including 33 overexpressed and 27 underexpressed. Overexpressed ASE genes included Keratin 15, Collagen IV/V, TRIM16, and Angiopoietin-1-like, which are associated with epithelial integrity, immune response, and tissue remodeling. Underexpressed ASE genes such as SOCS3, CSF3R, and Neutrophil cytosolic factor suggest individual variation in cytokine signaling and oxidative stress pathways. Conclusions: several ASE genes co-localized with previously identified QTLs for sea lice resistance, indicating that cis-regulatory variants contribute to phenotypic differences in parasite susceptibility. These results highlight ASE analysis as a powerful tool to identify functional regulatory elements and provide valuable candidates for selective breeding and genomic improvement strategies in aquaculture.

## 1. Introduction

Aquaculture is the fastest-growing sector of animal protein production globally, reaching 223.2 million tonnes in 2022 [1]. Among aquaculture species, salmonids represent a major economic driver, with growth rate, disease resistance, and carcass quality being primary traits targeted in selective breeding programs [2]. In Chile, harvest weight is the main selection criterion, typically around 3 kg for coho salmon and rainbow trout, and 4.5–5.5 kg for Atlantic salmon. However, parasitic infections such as those caused by *Caligus rogercresseyi* (sea lice) represent a major constraint to the sustainability and productivity of salmon aquaculture [3].

Control strategies have relied on antiparasitic treatments, though resistance has emerged [4], prompting increased interest in genetic resistance as a sustainable alternative [5]. Heritability estimates for resistance to sea lice in *Salmo salar* range from 0.12 to 0.32 [6,7,8], indicating sufficient genetic variation for improvement.

Sea lice impact growth performance and productivity, raising questions about the genetic relationship between growth and resistance. Favorable genetic correlations (e.g., *r_g_* = −0.32) suggest that selection for growth may reduce parasite burden [7]. Nevertheless, the effect of infestation on growth dynamics remains unclear. Growth under parasite challenge may represent a distinct trait from growth in uninfected conditions, aligning with the concepts of tolerance and resilience [9,10]. Understanding the genetic basis of these traits is crucial for developing robust breeding strategies that improve both productivity and disease resistance in salmon aquaculture.

Genome-wide association studies (GWASs) have been employed as a genomic approach to elucidate the genetic architecture underlying economically important traits in aquaculture species, including Atlantic salmon (*Salmo salar*) [11,12,13]. These studies have consistently demonstrated that growth-related traits are polygenic in nature, influenced by numerous loci each contributing small to moderate effects. Similarly, resistance to *Caligus rogercresseyi* has been characterized as a polygenic trait, with no loci of major effect detected to date [12,14,15]. Notably, Robledo et al. [16] identified three quantitative trait loci (QTLs) associated with sea lice resistance that collectively accounted for 7–13% of the genetic variance, although only one locus reached genome-wide significance.

Gene expression profiling technologies, such as RNA sequencing (RNA-seq) [17], offer a powerful tool to investigate host transcriptional responses under different experimental conditions or phenotypic groups. This approach allows for a comprehensive understanding of host–parasite interactions by identifying genes and molecular pathways involved in the response to infestation. Comparative transcriptomic studies between resistant and susceptible salmonid species, such as coho salmon (*Oncorhynchus kisutch*) or pink salmon (*O. gorbuscha*) versus Atlantic salmon, have demonstrated key differences in the activation of innate immune responses [18,19]. In resistant species, increased expression of pattern recognition receptors (PRRs), such as C-type lectins; proinflammatory cytokines, including interleukin-1β; and mechanisms related to iron sequestration or depletion have been reported [20].

Beyond interspecies comparisons, transcriptomic analyses of individuals with contrasting phenotypes can help reveal candidate genes involved in trait variation. However, studies examining differential gene expression between resistant and susceptible Atlantic salmon families in response to sea lice infestation remain limited. One study analyzing 32 immune-related genes indicated that resistant fish might avoid parasite-induced immunosuppression [21].

Additionally, allele-specific expression (ASE) has emerged as a complementary tool to study the functional effects of genetic variants in aquaculture species. ASE allows for the detection of differences in expression between paternal and maternal alleles within the same individual, which can reveal cis-acting regulatory variants that directly affect genes relevant to the immune response. In Atlantic salmon, for example, ASE has been used to identify functional variants in disease resistance-related genes such as *cd83* and *tlr5*, where one allele shows higher expression in more resistant individuals [16]. These analyses have proven useful in prioritizing variants in GWASs, providing a functional dimension that helps establish causal links between genotype and phenotype.

In rainbow trout, ASE has also been used to detect expression biases in genes involved in metabolism and immune response under thermal stress and pathogen exposure conditions [22]. Integrating ASE data with transcriptomics and functional genomics represents a promising approach to enhance the accuracy of genetic selection in aquaculture, facilitating the identification of robust biomarkers for resistance and tolerance.

Moreover, to date, no published studies have investigated whether genetic variants contribute to allelic-specific expression (ASE) associated with susceptibility to sea lice (*C.rogercresseyi*) infestation. This is particularly relevant for aquaculture breeding programs aiming to improve multiple traits simultaneously. Identifying pleiotropic loci or shared regulatory networks could enhance selection strategies by enabling genomic predictions that account for complex trait interdependencies. Thus, integrative approaches combining high-resolution genotyping, RNA-seq, ASE, and eQTL mapping hold great promise for unraveling the genetic basis of economically important traits in Atlantic salmon and other aquaculture species.

This study aims to advance the current understanding of the genetic mechanisms underlying host resistance to *C. rogercresseyi* in Atlantic salmon by identifying loci exhibiting allele-specific expression. The insights gained from this work have the potential to inform the implementation of marker-assisted and genomic selection strategies, contributing to the development of more resilient and productive breeding populations through the identification of functionally relevant genomic targets.

## 2. Materials & Methods

### 2.1. Animals

The challenge with *C. rogercresseyi* was conducted following the procedure described Yañez et al. [7]. A total of 1200 fish were distributed in a 7 m^3^ pond with saltwater at 10 ppt at the ATC Patagonia Technological Center. A sanitary check was performed prior to their entry into the experimental center. RT-PCR was conducted on 15 samples of kidney and liver tissue to verify that the animals were free of *Flavobacterium* p., Infectious Pancreatic Necrosis Virus (IPNv), *Piscirickettsia salmonis*, *Renibacterium salmoninarum*, and *Piscine orthoreovirus*.

A 15-day acclimatization period was implemented, during which salinity was gradually increased to 100% seawater. Acclimatization conditions were maintained at a temperature of 12 ± 1 °C, pH of 7.4 ± 0.1, and oxygen saturation of 98 ± 2.7%. Culture conditions, including temperature, oxygen, pH, and salinity, were monitored three times daily at 09:00, 12:30, and 16:30 using OxyG Handy Polaris portable equipment (Duotek Services SpA, Puerto Montt, Chile). Prior to the challenge test, a second measurement of body weight (CBW) and length (CBL) was recorded for each fish.

To perform the infestation, copepodites provided by the Crustacean Ecophysiology Laboratory of the Universidad Austral de Chile were used. Upon arrival at the ATC Patagonia Technological Center, copepodite cultures were checked for developmental stage, viability, and behavior. The copepodites were maintained at a temperature of 10 ± 0.5 °C. Infestation with *C. rogercresseyi* at the copepodite stage involved introducing 108,000 parasites (approximately 90 copepodites per fish) into the pond. The infestation process consisted of introducing the copepodites into the pond, kept in total darkness by covering it with a black tarp, and maintaining static water flow for 5 h. During this period, oxygen and temperature were continuously monitored and recorded, with manual oxygen supplementation as needed. After the infestation period, the water flow was gradually restored.

Four days post-challenge, 1157 fish that had successfully completed the acclimatization period were evaluated. Parasite counts were conducted, differentiating parasite load by location (body, fins, and total load), and individual data were collected for PIT tag readings, weight, and length. This procedure was repeated on days 10 and 15 post-infestation under the same experimental conditions. Fish were anesthetized using 20% benzocaine (20 mL/100 L of water) before sampling and were subsequently returned to their pond of origin.

Following the first infestation and parasite count, the fish were deloused by gradually reducing salinity over a 14-day period. Salinity was lowered to 0 ppt, maintained for 2–3 days, and then gradually increased back to full-strength seawater. This procedure effectively removed all attached parasites, as confirmed by visual inspection. A total of 29 fish mortalities were recorded following the first delousing procedure.

The second challenge was conducted under the same conditions and procedures as the first, 12 days after the conclusion of the previous challenge. This round included 1021 fish and resulted in 35 recorded mortalities. At the conclusion of the study, the fish were euthanized to assess variables relevant to the trial’s objectives, including parasite burden, body weight, and body length. Final body weight (FBW) and final body length (FBL) were recorded for each fish. Passive integrated transponder (PIT) tags were used to accurately link each individual to its respective phenotypic data.

Several standardized phenotypic definitions were used. Body weight was recorded at three key stages: initial body weight (IBW), measured at the beginning of the acclimation period; challenge body weight (CBW), recorded at the start of each experimental infestation; and final body weight (FBW), measured at the end of each challenge.

Parasite load was assessed through lice count (LC), which corresponded to the number of sea lice manually counted on the skin surface of each fish. Given that LC data typically exhibit a left-skewed distribution, log transformation is often applied to approximate normality and ensure that the assumptions of parametric models are met [23]. To account for host body size and surface area available for parasite attachment, lice density (LD) was calculated as (LC + 1) divided by the cubic root of the square of FBW, where FBW is expressed in grams. This surface area-corrected measure, proposed by [23], is defined as:

$$LD=\frac{LC+1}{\sqrt[{3}]{{FBW}^{2}}}$$

where FBW is the body weight (g) recorded at the end of the experimental challenge, and $∛{FBW}^{2}$ is an approximate measure of the fish’s surface area.

Log-transformed lice density (LogLD): To normalize lice density data, a natural logarithm transformation was applied, defined as:

$$LogLD={Log}_{e}\left(\frac{LC+1}{\sqrt[{3}]{{FBW}^{2}}}\right)$$

The LC value increased by 1 when individuals had no attached parasites, ensuring the correct application of the logarithmic transformation.

### 2.2. Transcriptome Sequencing

The RNA-seq experiment biopsies were taken from the skin near the caudal fins of 951 animals, of which 85 animals were selected for RNA-seq. These were chosen by the sum of parasite load corrected by their weight in all measurements, considering 43 with higher total load and 42 with lower total load, thus considering both extremes of sea lice load. Tissue samples were preserved in RNA Later and stored at −80 °C for total mRNA purification. They were purified from approximately 5 mg of tissue using a standard TRI reagent RNA extraction protocol. After RNA quality control, libraries were prepared using the Illumina Truseq mRNA library preparation kit protocol. The BGI company performed mRNA extraction and sequencing with 150-bp paired-end reads.

### 2.3. RNA-Seq Quality Control and Quantification

The quality of the RNA sequences was evaluated using FastQC v.0.11.5 (http://www.bioinformatics.babraham.ac.uk/projects/fastqc/ accessed on 1 April 2023). Residual adapter sequences were removed using Trimmomatic v.0.32 [24]. The filtered reads were aligned to the latest version of the Atlantic salmon reference genome, Ssal_v3.1 (GenBank accession: GCA_905237065.2), using the RNA-seq aligner STAR v2.7.11b [25]. Transcript abundance was quantified using RSEM v1.3.3 [26]. A reference transcriptome was generated using rsem-prepare-reference with the Atlantic salmon genome assembly (GenBank: GCA_905237065.2) and the corresponding gene annotation file in GFF format. RNA-Seq reads were aligned to this reference using the integrated STAR aligner within RSEM. Expression quantification was performed using the rsem-calculate-expression command with the --paired-end option. Gene-level expected counts were obtained from the .genes.results files and used as input for differential expression analysis with the DESeq2 [26] package in R.

After this, the GATK v4.5.0.0 module ASEReadCounter [27] was used to calculate the read count per allele for allele-specific expression analysis from the BAM files generated by alignment with STAR [25]. Using the allele-specific read count, differential gene expression (DEG) characterization was performed using the DESeq2 v1.48 R package [28]. To control false-discovery rates (FDR) in multiple hypothesis tests, *p*-values calculated by DESeq2 were adjusted. Subsequently, a list of differentially expressed transcripts with adjusted *p*-value less than 0.05 and log2FC (fold change in gene expression) > 1 for upregulated genes and log2FC < −1 for downregulated genes was generated.

The groups of downregulated and upregulated genes were used to calculate allele-specific expression (ASE), which was quantified through the differential abundance of allele copies of a transcript to identify putative regulatory polymorphisms acting in cis. The R package ASEP (Allele-Specific Expression Analysis in a Population, version 0.1.0) [29] was used. The “ASE_detection” function of the ASEP package was applied to detect significant ASE effects at the gene level (*p*-value < 0.05) within the population and grouped by upregulated and downregulated genes. Gene annotation of the identified genes was performed using the R package bioMart v2.65.0 [30], which uses the EMBL-EBI database, the European Bioinformatics Institute [31], and the Atlantic salmon reference genome, Ssal_v3.1.

## 3. Results

Fish were weighed and the numbers of attached sea lice counted on days 4, 10, and 15 of both the first and second challenge trials (Table 1). The average body weight prior to the first challenge was 168.64 ± 24.37 g, increasing to 170.56 ± 28.49 g at the end of the first challenge and reaching 184.04 ± 31.71 g by the conclusion of the second challenge.

Regarding sea lice counts, an average of 18.99 ± 8.67 parasites per fish was recorded at the end of the first challenge and 17.41 ± 6.75 at the end of the second challenge. Infestation success was calculated as the average proportion of parasites successfully infesting a host relative to the total number of parasites available per host during the infestation period [32]. This metric was evaluated at the end of each challenge, yielding infestation success rates of 47.46% and 43.51% for the first and second challenges, respectively.

**Figure 1:**
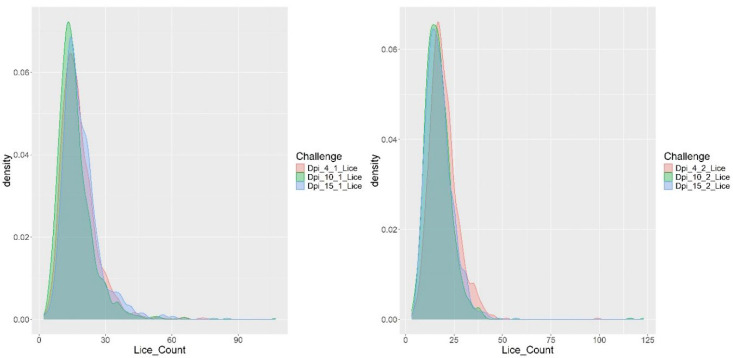
Density plot for the first and second challenges, showing parasite counts on days 4, 10, and 15.

The distribution of parasite burden throughout the infestation is illustrated in Figure 1. A slight increase in the number of attached parasites was observed during the first challenge, whereas a decrease over time in attached parasite numbers was evident during the second challenge.

Table 1 summarizes the descriptive statistics for fish weight, length, and sea lice counts recorded at different time points during two consecutive infestation challenges. Fish weight increased steadily from an average of 168.64 g pre-challenge to 184.04 g at the end of the second challenge, indicating ongoing growth throughout the experimental period. Length measurements remained relatively stable, with low variability (CV ≈ 4.8%). Sea lice counts showed a moderate decrease from day 4 to day 15 post-infestation in both challenges, with a more pronounced reduction observed in the second challenge (from 19.64 to 17.41 lice per fish). Parasite burden variability was considerable (CV > 38%), highlighting individual differences in host susceptibility or parasite attachment success. The consistent parasite loads alongside increasing host size suggest a dynamic interaction influenced by host immune responses and possibly environmental or stress-related factors. Overall, these results underscore the importance of repeated longitudinal measurements for accurately characterizing infestation dynamics and host resilience in Atlantic salmon.

### 3.1. RNA-Seq

RNA-seq reads from 85 samples were aligned to the *S. salar* reference genome (Ssal_v3.1) using STAR v2.7.10a with default parameters and the --quantMode TranscriptomeSAM option enabled to generate transcriptome-aware alignments for downstream quantification. On average, each sample yielded approximately 36.1 million paired-end reads (SD = 66,703; range: 36.0–36.3 million).

The alignment was highly efficient, with a mean of 81.2% of reads mapping uniquely to the reference genome (range: 73.8–87.7%; SD = 3.15) and an additional 6.6% mapping to multiple loci (range: 4.85–8.51%; SD = 0.77). The average mismatch rate per base was 0.31% (range: 0.23–0.37%; SD = 0.035), indicating high sequence quality and mapping accuracy.

Splice junction analysis revealed an average of 21.1 million spliced alignments per sample (SD = 1.61 million; range: 18.0–25.9 million), of which 94.4% corresponded to annotated splice sites, with a mean of 19.9 million annotated junctions per sample (SD = 1.65 million; range: 16.8–25.1 million).

These results confirm that the RNA-seq data were of high quality and that the STAR-based alignment against the *S. salar* Ssal_v3.1 genome provided accurate and comprehensive coverage of the transcriptome.

### 3.2. Allele-Specific Expression Analysis

To detect and quantify allele-specific expression (ASE), we employed the R package Allele-Specific Expression in a Population (ASEP) [29], which enables population-level modeling of ASE. This approach allows for the identification of genes exhibiting biased allelic expression across individuals and facilitates the detection of specific alleles potentially associated with phenotypic traits of interest.

**Figure 2:**
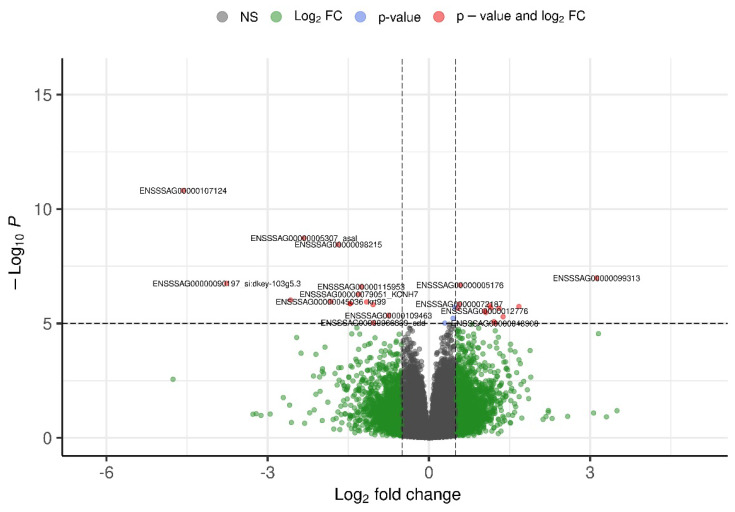
Volcano plot showing the expression variation of 69,389 evaluated genes. The *y*-axis indicates the −log of the *p*-value, and the *x*-axis indicates the log fold change.

To complement the ASE analysis, a differential expression study was conducted to identify genes exhibiting significant up- or downregulation. This information was integrated with ASE results to prioritize candidate genes and alleles potentially involved in trait regulation. A total of 69,389 genes were considered in the transcriptomic matrix. From this dataset, 171 genes were selected based on a nominal p-value < 0.05 and a false discovery rate-adjusted p-value (FDR-adjusted p-value, padj) < 0.02. These genes were used for subsequent analyses. The selected genes were classified into two groups according to their expression patterns: 104 genes showed significant upregulation, while 67 genes exhibited downregulation (Figure 2).

### 3.3. Candidate Genes

The candidate genes exhibiting significant allele-specific expression (ASE) with concurrent overexpression during sea lice infestation in Atlantic salmon are shown in Table 2. A total of 33 genes showed robust ASE signals (*p*-value < 0.05), highlighting their potential regulatory modulation in response to parasite challenge. Notably, genes involved in structural integrity and immune response were prominently represented. For example, two paralogs of *Keratin 15* (*ENSSSAG00000045136* and *ENSSSAG00000048512*; *p* = 0.003), *Collagen type IV alpha 5* (*ENSSSAG00000064682*), and *Collagen type V alpha 3a* (*ENSSSAG00000090709*) likely contribute to mucosal barrier maintenance and skin remodeling. Immune-related candidates include *Tripartite motif-containing protein 16-like* (*ENSSSAG00000104872*), a known innate immunity regulator, and Serine protease 35 (*ENSSSAG00000119277*), potentially involved in antimicrobial defense. Other notable genes include *Fibronectin type III* domain containing 1 (*ENSSSAG00000008223*), implicated in cell adhesion, and Angiopoietin-1-like (*ENSSSAG00000012776*), which may participate in vascular remodeling and tissue repair. Several novel and uncharacterized genes also demonstrated significant ASE, warranting further investigation. Collectively, these findings support the role of cis-regulatory variation in modulating key biological pathways underlying host resistance to *C. rogercresseyi* and provide valuable targets for functional validation and selective breeding programs.

In contrast to the upregulated ASE candidates, a distinct set of 27 underexpressed genes also exhibited significant allelic imbalance, as summarized in Table 3. These genes were predominantly associated with nucleolar function, RNA metabolism, and immune regulation. Several transcripts encoding nucleolar components, including *NOP14 nucleolar protein homolog* (*ENSSSAG00000002452*), *Nucleolar protein 8* (*ENSSSAG00000060839*), *Nucleolar protein 11* (*ENSSSAG00000064522*), and *MIF4G domain-containing nucleolar protein 1* (*ENSSSAG00000068370*), displayed strong ASE signals, suggesting reduced ribosomal biogenesis and global protein synthesis during advanced stages of infestation. Immune-related genes such as *Suppressor of cytokine signaling 3* (*SOCS3*, *ENSSSAG00000067044*) and *Colony-stimulating factor 3 receptor* (*CSF3R*, *ENSSSAG00000072535*) were also significantly underexpressed with ASE, pointing to potential genotype-dependent attenuation of cytokine signaling pathways. The downregulation of *Neutrophil cytosolic factor* (*ENSSSAG00000079828*), a component of the NADPH oxidase complex, may impair reactive oxygen species (ROS) generation, further supporting the hypothesis of host tolerance mechanisms aimed at minimizing immunopathology. Additionally, transcripts related to extracellular matrix degradation, such as *Matrix metalloproteinase 9* (*ENSSSAG00000069874*) and *Collagenase 3* (*ENSSSAG00000070495*), were suppressed, indicating a temporal shift in tissue remodeling responses. The ASE landscape of underexpressed genes complements the profile of overexpressed loci, underscoring a coordinated regulatory program involving both activation and repression of critical biological processes during sea lice infestation. The presence of ASE in genes with established or plausible roles in resistance mechanisms supports their candidacy as functional targets for selective breeding and molecular validation.

## 4. Discussion

Two consecutive sea lice (*C. rogercresseyi*) challenge trials were conducted, yielding valuable insights into parasite burden and growth dynamics in Atlantic salmon over time. Infestation success rates of 47% and 43% were recorded across the two trials, reflecting consistently high infection efficiency [32]. As shown in Figure 1, a shift in infestation dynamics occurred during the second challenge, characterized by a reduction in attached parasites. This decline was likely due to elevated stress levels in the fish [33], damaged mucosal surfaces [34], and decreased host attractiveness [35].

The integration of allele-specific expression (ASE) analysis with differential gene expression profiling revealed a suite of genes potentially modulated by cis-regulatory variants and involved in Atlantic salmon resistance to sea lice. Notably, 33 overexpressed and 27 underexpressed genes demonstrated significant ASE (*p*-value < 0.05), providing strong evidence for the contribution of regulatory variation to host–parasite interactions.

Among the overexpressed genes, several encode structural and immune-related proteins previously implicated in parasite resistance. Two paralogs of *Keratin 15* (*ENSSSAG00000045136* and *ENSSSAG00000048512*; *p* = 0.003), together with *Collagen type IV alpha 5* (*ENSSSAG00000064682*) and *Collagen type V alpha 3a* (*ENSSSAG00000090709*), exhibited robust ASE signals, supporting their involvement in mucosal barrier integrity and skin remodeling upon ectoparasite attachment [31,36]. Tripartite motif-containing protein 16-like (*TRIM16*, *ENSSSAG00000104872*), a known modulator of innate immunity and cell proliferation [37], also displayed strong ASE (*p* = 0), reinforcing its candidacy as a master regulator of resilience to sea lice.

Other upregulated ASE genes include Angiopoietin-1-like (*ENSSSAG00000012776*), potentially involved in vascular remodeling and tissue repair [38], and *Matrix metallopeptidase 28* (*ENSSSAG00000076502*), consistent with extracellular matrix (ECM) degradation and tissue regeneration. The presence of *Fibronectin type III* domain-containing 1 (*ENSSSAG00000008223*) and Adhesion G-protein-coupled receptor A3 (*ENSSSAG00000069698*) suggests active modulation of cell adhesion and signaling, processes previously associated with parasite defense [39]. Enzymes like D-amino acid oxidase (*ENSSSAG00000063640*) and *Serine protease 35* (*ENSSSAG00000119277*) may contribute to antimicrobial activity and skin defense, while *IGFBP2a* (*ENSSSAG00000056022*) might link growth immune trade-offs during parasite challenge [40].

Conversely, the underexpressed ASE genes include numerous transcripts involved in nucleolar function, RNA processing, and innate immune signaling. The downregulation of *Matrix metalloproteinase 9* (*ENSSSAG00000069874*) and *Collagenase 3* (*ENSSSAG00000070495*) may reflect a context-specific suppression of ECM turnover during later stages of infestation, contrasting with earlier reports [18]. Several nucleolar proteins with ASE, such as NOP14, Nucleolar protein 8, and MIF4G domain-containing protein 1, suggest an attenuated ribosome biogenesis and translational response under high parasitic burden.

Importantly, immune-related genes such as *Suppressor of cytokine signaling 3* (*SOCS3*) (*ENSSSAG00000067044*) and Colony-stimulating factor 3 receptor (CSF3R) (*ENSSSAG00000072535*) were significantly underexpressed with ASE, indicating a possible genotype-dependent suppression of cytokine signaling pathways. These genes have been previously linked to granulopoiesis and inflammatory regulation [41,42], and their repression could represent a tolerance mechanism aimed at limiting immunopathology. Likewise, Neutrophil cytosolic factor (*ENSSSAG00000079828*) showed consistent ASE and downregulation, which may impact reactive oxygen species (ROS) generation.

The ASE landscape highlights the role of cis-regulatory variants in modulating gene expression in key biological processes such as skin barrier maintenance, ECM remodeling, innate immunity, and cellular stress response. Many ASE genes detected in this study, such as *TRIM16*, *Keratin 15*, *CSF3R*, and *SOCS3*, map near previously identified QTLs associated with sea lice resistance and host resilience traits [8,36]. Their co-localization supports a model in which expression-modulating variants contribute causally to observed phenotypic variance, providing targets for genomic selection or functional validation.

Future work should focus on fine-mapping cis-regulatory elements affecting ASE genes and validating their functional roles via genome editing or transgenic approaches. The integration of ASE with eQTL and GWAS data continues to represent a powerful strategy for dissecting complex traits such as parasite resistance in aquaculture species.

## 5. Conclusions

This study integrated transcriptomic profiling, variant discovery, and allele-specific expression (ASE) analysis to investigate the role of cis-regulatory variation in Atlantic salmon. Several overexpressed ASE genes were functionally relevant, including *Angiopoietin-1-like*, *IGFBP2a*, *Collagen IV* and V isoforms, *TRIM21-like*, keratins (e.g., KRT15), metalloproteinases (e.g., *MMP28*), and adhesion molecules (, *ADGR-A3*), highlighting coordinated regulation of tissue integrity, growth, and immune function. Among underexpressed ASE genes, key candidates such as *ABCA1*, *SOCS3*, *MMP9*, and *Collagenase 3* were identified, alongside nucleolar and RNA-processing genes, suggesting transcriptional control mechanisms. This integrative approach enables the prioritization of functional variants and candidate genes for host resistance and performance traits. These findings advance our understanding of the genetic architecture of complex traits in salmonids and highlight the utility of ASE analyses in functional genomics and selective breeding. Further validation across diverse populations will help link these regulatory variants to economically important phenotypes.

## Figures and Tables

**Table 1:** Summary statistics of fish weight, length, and parasite counts during pre-challenge and days post-infestation (DPI) in two consecutive sea lice challenges. DPI, days post-infestation; N, numbers of animals; SD, standard deviation; CV, coefficient of variation.

Trait	N	Mean	SD	Med.	Min	Max	CV (%)
Weight Pre-challenge	1126	168.6	24.4	165	108	271	14.5
Length Pre-challenge	1126	24.3	1.16	24.3	15.6	29.5	4.8
DPI4_1 Parasites	1127	18.0	8.2	17	2	75	45.8
DPI10_1 Parasites	1122	16.4	8.1	15	4	107	49.2
DPI15_1 Parasites	1019	19.0	8.7	17	2	85	45.7
DPI4_1 Weight	1127	169.1	24.1	167	108	278	14.3
DPI10_1 Weight	1122	166.9	23.8	165	104	276	14.3
DPI15_1 Weight	1019	170.6	28.5	169	16.3	402	16.7
DPI4_2 Parasites	992	19.6	7.5	19	4	99	38.2
DPI10_2 Parasites	978	17.0	7.8	16	3	123	45.5
DPI15_2 Parasites	952	17.4	6.8	16	4	57	38.8
DPI4_2 Weight	992	179.1	29.9	178	85	312	16.7
DPI10_2 Weight	978	188.1	32.8	186	94	340	17.4
DPI15_2 Weight	952	184.0	31.7	184	5	320	17.2

**Table 2:** Overexpressed genes that exhibit ASE along with their respective *p*-values.

Gene	*p*-Value	Description
*ENSSSAG00000008223*	0	Fibronectin type III domain containing 1
*ENSSSAG00000012776*	0.005	Angiopoietin-1-like
*ENSSSAG00000035308*	0	Membrane-associated ring-CH-type finger 3
*ENSSSAG00000045136*	0.003	Keratin 15
*ENSSSAG00000048512*	0.003	Keratin 15
*ENSSSAG00000054925*	0	Myristoylated alanine-rich protein kinase C substrate
*ENSSSAG00000056022*	0.045	Insulin-like growth factor binding protein 2a
*ENSSSAG00000063640*	0	D-amino acid oxidase
*ENSSSAG00000064326*	0	GULP PTB domain-containing engulfment adaptor 1a
*ENSSSAG00000064378*	0	novel
*ENSSSAG00000064682*	0	Collagen type IV alpha 5
*ENSSSAG00000065229*	0.005	Novel gene
*ENSSSAG00000069698*	0	Adhesion G protein-coupled receptor A3
*ENSSSAG00000070277*	0	Beta 3-glucosyltransferase a
*ENSSSAG00000070579*	0	Disintegrin and metalloproteinase with thrombospondin motifs 12
*ENSSSAG00000073657*	0.001	LOC106569355
*ENSSSAG00000076502*	0.035	Matrix metallopeptidase 28
*ENSSSAG00000076511*	0.004	EGF-like domain multiple 6
*ENSSSAG00000083329*	0	LOC106612420
*ENSSSAG00000085545*	0	Novel gene
*ENSSSAG00000087971*	0.003	Synaptotagmin XII
*ENSSSAG00000090709*	0.001	Collagen type V alpha 3a
*ENSSSAG00000094685*	0.002	Sperm acrosome-associated 4-like
*ENSSSAG00000097547*	0	Novel gene
*ENSSSAG00000102125*	0	Osteoglycin
*ENSSSAG00000104092*	0.008	Novel gene
*ENSSSAG00000104872*	0	Tripartite motif-containing protein 16-like
*ENSSSAG00000104937*	0	Iodothyronine deiodinase 1
*ENSSSAG00000109080*	0	CD248 molecule, endosialin a
*ENSSSAG00000111485*	0	Novel gene
*ENSSSAG00000118058*	0	Myosin-7
*ENSSSAG00000119277*	0	Serine protease 35
*ENSSSAG00000122110*	0.01	E3 ubiquitin-protein ligase TRIM21-like

**Table 3:** Subexpressed genes that exhibit ASE along with their respective *p*-values.

Gene	*p*-Value	Description
*ENSSSAG00000000392*	0	RNA exonuclease 5
*ENSSSAG00000002452*	0	NOP14 nucleolar protein homolog (yeast)
*ENSSSAG00000003919*	0.016	Novel gene
*ENSSSAG00000004485*	0	Immunoglobulin-like and fibronectin type III domain-containing 1
*ENSSSAG00000008409*	0.001	Cell death-inducing DFFA like effector b
*ENSSSAG00000010799*	0	Novel gene
*ENSSSAG00000015527*	0	Palmitoyltransferase
*ENSSSAG00000018923*	0	Phospholipid-transporting ATPase ABCA1
*ENSSSAG00000041926*	0	Phospholipid-transporting ATPase ABCA1
*ENSSSAG00000045036*	0	Keratin 99
*ENSSSAG00000049698*	0	RNA binding motif protein 34
*ENSSSAG00000060839*	0	Nucleolar protein 8
*ENSSSAG00000064522*	0	Nucleolar protein 11
*ENSSSAG00000066839*	0.001	Cytidine deaminase
*ENSSSAG00000067044*	0	Suppressor of cytokine signaling 3
*ENSSSAG00000068370*	0	Nucleolar protein with MIF4G domain 1
*ENSSSAG00000068818*	0	TRNA methyltransferase 2 homolog A
*ENSSSAG00000069874*	0.016	Matrix metalloproteinase 9
*ENSSSAG00000070332*	0	AMP-activated alpha 1 catalytic subunit
*ENSSSAG00000070495*	0	Collagenase 3
*ENSSSAG00000071944*	0	Nle1
*ENSSSAG00000072535*	0.024	Colony-stimulating factor 3 receptor (granulocyte)
*ENSSSAG00000079794*	0	Novel gene
*ENSSSAG00000079828*	0	Neutrophil cytosolic factor
*ENSSSAG00000080634*	0	Deoxynucleotidyltransferase, terminal, interacting protein 2
*ENSSSAG00000090197*	0.039	Zona pellucida-like domain-containing protein 1
*ENSSSAG00000117724*	0	Guanine deaminase

## Data Availability

The data that support the findings of this study are the property of AquaGen Chile, and restrictions apply to their availability; they were used with permission for the current study. Therefore, the data are not deposited in a public repository but can be accessed under agreement with AquaGen Chile.
